# Some Diseases of the Male Genital System

**Published:** 1908-03-07

**Authors:** 


### SOME DISEASES OF THE MALE GENITAL SYSTEM.

### X.—TUBERCULOSIS OF THE TESTIS AND EPIDIDYMIS (*Continued*).

From the previous articles it will have been seen that there are points in the disease which should make the diagnosis of a tuberculous testis an easy matter. The most important of these are its tendency to start in the epididymis rather than in the body of the testis itself, the presence of hard nodular swellings in the epididymis and the subsequent appearance of soft caseating areas which eventually involve the skin and come to the surface, leaving a discharging sinus. The diagnosis may be made certain in the later stages by a microscopical examination of the discharging matter, or of the granulation tissue, which can be scraped from the walls of the sinus. In the former it may be possible to demonstrate the tubercle bacillus, or in the latter typical tuberculous giant-cells. Either of these, of course, clinches the diagnosis. In spite of this, the diagnosis of early cases may be difficult, for the incontrovertible evidence afforded by microscopical examination is not obtainable.

It has already been said that tuberculosis may follow on a gonorrhœal epididymitis, and in the early stages the two conditions present many points of similarity, so that it is not easy to say when one process passes into the other. The only points on which reliance can be placed are the history of a recent or remote attack of gonorrhœa and the presence of a discharge from the meatus. If such a history can be obtained the case is probably one of gonorrhœal epididymitis. Orchitis due to syphilis may occasionally mislead one. But in a typical case the appearances presented are quite distinctive. In syphilis the body of the testis is more affected than the epididymis; it is enlarged, hard, and rounded. It is more common to find both testicles simultaneously affected than in tuberculous disease, in which the second is generally involved some time after the first. Testicular sensation is quite absent in syphilitic orchitis, as evidenced by the fact that no pain is evinced by firm pressure of the organ: in tuberculosis, even in the late stages, some portion of the testis can be found which retains sensation. The cord is not thickened at first; later it may undergo a regular enlargement to compensate for the increased weight; and, finally, although a hydrocele may be associated with any disease of the testis, it is a more common phenomenon in syphilitic than in other affections. A history of syphilis or other signs of the disease will help to corroborate the diagnosis. It should, therefore, be an easy matter to distinguish between the two causes. Unfortunately, cases of testicular disease have a habit of departing somewhat widely from the text-book descriptions, and reliance has to be placed on, perhaps, only one of these differential signs. Sarcoma of the testis present points of similarity, but may be distinguished from tuberculosis by its rapid growth, by the thickening of the lymphatics of the cord, and by the presence of enlarged lumbar glands which may be palpable through the anterior abdominal wall. Hydrocele in association with sarcoma is not common. It resembles a tuberculous testis in its irregular outline and in being painful.

The treatment of "galloping phthisis" of the testis is immediate castration with removal of as much of the cord and vas deferens as can be isolated: expectant treatment is useless, since the very nature of the process is an indication that the patient has no resistance to tuberculous infection. In the chronic form palliative treatment may be tried. It consists more in looking after the patient's general health, so as to increase his natural resistance to the tubercle bacillus, than in adopting any local measures, apart from protecting the testis from injury by applying a suspensory bandage. In a small proportion of cases the disease disappears, but in the greater number it continues its course unchecked.

In this case some form of operative treatment must be undertaken, and the question arises as to whether the testis itself can be saved. It is important to do this whenever it is possible, because it has been shown that it is the source of an internal secretion which is of value to the individual. If therefore, the epididymis alone is involved and there is no evidence of a central abscess in the body of the testis, the operation of epididymectomy may be performed. An incision is made over the affected testis and the epididymis carefully separated from it. The important point of the operation is to avoid damage to the vessels which lie on its inner side. If these are injured the blood-supply to the testis will be interfered with, and castration will be necessary later. After separating the epididymis from the testis it is removed with as much of the vas as can be isolated. This proceeding may or may not result in the removal of all the disease present, but even if it does not it gives the testicle a chance of recovering itself and resisting further attacks.

If the body of the organ is already involved and is the seat of one or more abscesses the outlook is not so hopeful; but unless it is hopelessly disorganised the abscesses may be scraped on the chance that after the removal of the main foci of disease the smaller ones that are left will clear up of their own accord. Unfortunately one's clinical experience compels one to admit that this rarely occurs, and the usual ending is castration. Castration, as performed for tuberculosis, does not differ from the set operation, except that it is necessary to remove as much of the cord and vas deferens as can be isolated. If a high incision is made over the external abdominal ring this can be done as far up as the internal ring. If at the time of operation an abscess has come to the surface and involved the skin, the affected portion of the scrotum must be included in the incision and removed. Some surgeons have advocated an even more radical operation by means of two incisions, scrotal and perineal, through which the whole cord of the affected side, including the vesicula seminalis can be taken away. But this is not a sound surgical procedure. The perinæum should only be opened to drain an abscess involving the prostate or vesicula.

